# ADJUSTING FOR COUNTRY-LEVEL VARIATION IN DEMENTIA PREVALENCE WITH CLASSIFICATION ALGORITHMS IN SHARE

**DOI:** 10.1093/geroni/igac059.1873

**Published:** 2022-12-20

**Authors:** Matthias Klee, Kenneth Langa, Anja Leist

**Affiliations:** University of Luxembourg, Esch-sur-Alzette, Not Applicable, Luxembourg; University of Michigan, Ann Arbor, Michigan, United States; University of Luxembourg, Esch-sur-Alzette, Grevenmacher, Luxembourg

## Abstract

Background. Population-level dementia prevalence depends on societal factors and individual-level risk and protective factors. To improve our understanding of how these factors interact, we can use cross-national surveys such as the Survey of Health, Ageing and Retirement in Europe (SHARE). However, in absence of validated cognitive assessments, adjusting for underdiagnosis of dementia is needed. The present study sought to explore the usefulness of the Langa-Weir and alternative algorithms to detect probable dementia while accounting for country-level variation in estimated prevalence and underdiagnosis of dementia.Method. Data from 57,880 respondents aged 60 years and older to wave 7 of SHARE (2017) with non-missing data on variables related to sociodemographics and cognition were used. Adaptations of the Langa-Weir classification algorithm were compared to a weighted logistic regression model and an XGBoost classifier applying the synthetic minority oversampling technique. Different specifications of algorithms were tested globally and for individual countries and compared with the World Alzheimer’s Report (2015)’s country-level projections of dementia prevalence for 2018. Results. All algorithms accurately classified self-reported diagnosis of dementia (accuracy = 0.90-0.96), with the Langa-Weir classification based on recall and a cutoff reflecting country-specific prevalence outperforming other algorithms regarding compensation of underdiagnosis. Algorithmically detected probable dementia is associated with newly self-reported dementia diagnosis, drop-out and death two years later. Discussion. Identifying probable dementia through classification algorithms can increase statistical power and improve validity in cross-national investigations. Further research is needed to replicate the findings in validated cognitive assessments and to identify causes of cross-national variation in dementia underdiagnosis.

